# Cysteine-Mediated Root Growth Promotion in Strawberry (*Fragaria × ananassa*) Induced by *TgSWO*-Overexpressing *Trichoderma*

**DOI:** 10.3390/microorganisms13071480

**Published:** 2025-06-26

**Authors:** Xiaohui Meng, Yuanhua Wang, Xu Zhang, Hongjun Yang, Yilei Lu, Ye Xu, Xiong Zhang, Zhiming Yan

**Affiliations:** 1 Department of Agronomy and Horticulture, Jiangsu Vocational College of Agriculture and Forestry, Jurong 212400, China; xiaohuimeng769@163.com (X.M.); zhangxu@jsafc.edu.cn (X.Z.); hjyang@jsafc.edu.cn (H.Y.); lylacscy@outlook.com (Y.L.); yexu232220@163.com (Y.X.); 2 Engineering and Technical Center for Modern Horticulture, Jurong 212400, China; wangyuanhua@jsafc.edu.cn; 3 The Key Laboratory of Biology and Genetic Improvement of Oil Crops, The Ministry of Agriculture and Rural Affairs of the PRC, Oil Crops Research Institute, Chinese Academy of Agricultural Sciences, Wuhan 430062, China

**Keywords:** *Trichoderma guizhouense*, TgSWO, cysteine, strawberry, root growth promotion

## Abstract

Strawberry (*Fragaria × ananassa*) is a globally important economic crop valued for its nutritional and commercial significance. However, its growth is frequently challenged by various biotic and abiotic stresses. To enhance strawberry root development and resilience, we engineered a *Trichoderma guizhouense* NJAU4742 strain to overexpress the *TgSWO* gene, which encodes a plant cell-wall-loosening protein known to facilitate fungal penetration and colonization. Strawberry seedlings treated with the *TgSWO*-overexpressing *T. guizhouense* NJAU4742 strain (S-OE) exhibited significant improvements in shoot and root fresh weights, root surface area, and number of root tips, showing 1.37- to 2.00-fold increases compared with the strawberry seedlings inoculated with the wild-type *T. guizhouense* NJAU4742 (S-WT) and 2.00- to 3.44-fold increases compared with the uninoculated strawberry seedlings (S-CK). Field-emission scanning electron microscopy (SEM) of the S-OE roots revealed denser hyphal colonization. Transcriptome analysis of S-OE showed a decrease in genes related to defense and detoxification, while genes for cell-wall growth and hormone signaling increased, shifting focus from defense to growth. Metabolomic profiling identified cysteine as a key metabolite associated with induced growth, which was further validated through exogenous cysteine application experiments. This study highlights the potential of genetically enhanced *Trichoderma* for improving strawberry growth and provides new insights into root–microbe interactions and metabolite-mediated plant development.

## 1. Introduction

Strawberry (*Fragaria × ananassa*), as a globally popular economic crop, holds a significant position in the fruit market due to its rich vitamin C content, abundant antioxidants, and unique sweet flavor. It is not only favored by consumers but also contributes substantially to the agricultural economy [1,2]. Recent statistics indicate that the global strawberry cultivation area and yield have continued to increase, enhancing its economic value and social influence. However, strawberry cultivation faces numerous challenges. Biotic stresses, such as root rot and wilt caused by soil-borne pathogens, like *Fusarium* and *Phytophthora*, pose serious threats to plant health, potentially leading to substantial yield losses or even complete crop failure. Abiotic stresses, including soil nutrient imbalances, drought, and low temperatures, can further hinder fruit development and sugar accumulation, ultimately reducing fruit quality and market value [3]. To address these challenges, the application of plant growth-promoting microorganisms (PGPMs) has emerged as a promising strategy in modern agriculture [4]. Among them, *Trichoderma* spp. is particularly notable for their multifaceted mechanisms of action. *Trichoderma* can promote strawberry growth by producing plant hormones and improving the root microenvironment while also activating plant defense responses, enhancing resistance to both biotic and abiotic stresses and inhibiting soil-borne pathogens. Consequently, the use of *Trichoderma* has become an important biological approach to securing high-quality, high-yield strawberry production, offering broad prospects for research and practical application [5,6].

*Trichoderma* species (spp.) are well known for their ability to colonize plant roots, forming a symbiotic relationship that benefits the plant [7,8]. These fungi promote plant growth through various mechanisms [6,9,10]. For instance, they form biofilms in the rhizosphere and secrete extracellular polysaccharides, which enhance root development and nutrient absorption [11]. Additionally, *Trichoderma* species inhibit soil-borne pathogens, such as *Fusarium* and *Fusarium* wilt, by competing for nutrients and producing antibiotics, including green anisopliae, while also inducing plant systemic resistance [12]. Furthermore, *Trichoderma* secretes cellulase and hemicellulase to degrade refractory organic matter in the soil, releasing essential nutrients like nitrogen and phosphorus [13,14]. It also directly influences plant cell division and elongation by synthesizing hormone analogs, such as gibberellin and cytokinin [9,15]. In addition, cysteine can significantly enhance the growth of filamentous fungi and stimulate the secretion of lignocellulose-degrading enzymes, including cellulases, xylanases, and β-glucosidases [16,17]. These enzymes promote efficient degradation of plant cell wall components, facilitating hyphal penetration, colonization, and nutrient cycling in the rhizosphere [18,19].

These processes undoubtedly involve the participation of proteins encoded by multiple genes within *Trichoderma*, including various plant cell-wall-degrading enzymes, proteases, and proteins involved in plant hormone synthesis [8]. The I-type chitinase gene (*TasHyd1*) in *Trichoderma asperellum* encodes a protein that enhances plant defense mechanisms by strengthening attachment to the root surface, thus preventing damage from pathogenic microorganisms [20]. The endopolygalacturonase gene *ThPG1* in *Trichoderma harzianum* contributes to internal root colonization and promotes the degradation of root cell walls, which enhances root growth and improves soil properties [21]. Thus, alteration of these fungal genes may show a significant effect on the promotion of plant growth. Indeed, our previous study identified that the expansin-like protein TgSWO, secreted by *Trichoderma guizhouense* NJAU4742, could effectively promote colonization by *Trichoderma* of cucumber root systems and promote growth in cucumber root systems [6]. However, this study focused solely on the physiological responses of cucumber, without addressing the molecular basis or the interaction mechanisms between plants and roots. Furthermore, the potential application of *TgSWO* overexpression in other plants remains unclear.

Here, this study focuses on the key *TgSWO* gene in *Trichoderma* and employs gene overexpression technology as the primary approach to systematically investigate its role in reshaping strawberry root morphology. Microscopic imaging and morphological analyses could be used to accurately quantify changes in root traits, while metabolomics and related techniques could be applied to identify key metabolites involved in *Trichoderma–*strawberry interactions. Furthermore, the role of these metabolites in facilitating fungal colonization of strawberry roots is verified. Ultimately, the comprehensive effects of *TgSWO* overexpression on strawberry growth and development can be systematically evaluated. The findings of this study are expected to fill critical gaps in the understanding of the molecular mechanisms by which *Trichoderma* promotes strawberry growth and provide essential theoretical and technical support for the targeted improvement of *Trichoderma* strains, development of novel biological agents, and advancement of green and efficient strawberry production.

## 2. Materials and Methods

### 2.1. Fungal Cultivation

*Trichoderma guizhouense* NJAU4742 (CGMCCNO.12166, China Microbial Culture Collection Committee General Microbiology Center) was stored in Jiangsu Key Laboratory for Organic Solid Waste Utilization. The strain has been genetically identified, and the genome sequence is publicly available in the NCBI GenBank database under BioProject accession number: PRJNA314460. The strain was grown in potato dextrose agar (PDA) medium (Difco Laboratories, Detroit, Michigan), and the conidial suspension of the *T. guizhouense* NJAU4742 strain was prepared as described by Meng et al. [6].

### 2.2. Treatment of Strawberry with T. guizhouense NJAU4742

For the tissue-cultured seedling experiment, 15-day-old tissue-cultured strawberry seedlings were inoculated with the *T. guizhouense* NJAU4742 wild-type (WT) strain and *TgSWO*-overexpressing strain (OE) at a concentration of 10^5^ spores/mL and grown under controlled conditions at room temperature. After 30 days, the root development was observed. The root system images were captured using an Epson Perfection V700 Photo scanner (SEIKO EPSON corp., Suwa, Japan).

For the field-based strawberry experiment, the following three groups were established: overexpression fungus group, normal fungus group, and control group. Prior to transplantation, the roots of the strawberry seedlings were dipped in a transgenic bacterial solution (at a concentration of 10^5^ spores/mL) for planting, and the roots were irrigated every 15 days during the growth period. After 4 months, the observed indicators, including plant height, number of runners, and fresh weight, were analyzed.

### 2.3. Root Colonization of NJAU4742 via Quantitative PCR

Strawberry seedlings with uniform growth were inoculated with spores (a final concentration of 10^5^ germinated spores/mL) from the WT and OE strains of *Trichoderma*. The seedlings continued to grow for 7 days under greenhouse conditions. Total RNA came from the *Trichoderma* mycelium or was extracted as described in [6]. Quantitative PCR was employed to assess the *Trichoderma* colonization in the root systems of the different treatments. The UTF and ITS2P primers were used to amplify 158 bp fragments from the ITS region.

### 2.4. Observation of Root Structure via SEM

The strawberry roots were then divided into two sections. One section consisted of the root tips, which were cut to a length of approximately 5 mm from the root crown, while the other section consisted of the root hairs. The roots were immersed in a 2.5% (*v*/*v*) glutaraldehyde solution and stored at 4 °C. Root structures were observed using a light microscope (BX53, OLYMPUS, Tokyo, Japan) and a scanning electron microscope (ESEM, XL-30, Philips, Amsterdam, The Netherlands) to examine root morphology and structure [22].

### 2.5. Transcriptome Analysis

Strawberry seedlings (15 days old) cultured under three conditions (i.e., uninoculated, WT inoculated, and OE inoculated) were harvested, with the roots collected and flash-frozen in liquid nitrogen. Total RNA was extracted using a commercial RNA extraction kit, and the RNA’s integrity was verified via 1% agarose gel electrophoresis and a NanoDrop spectrophotometer [23]. High-quality RNA samples were subjected to transcriptome sequencing on an Illumina NovaSeq 6000 platform (Majorbio, Shanghai, China) to generate 150 bp paired-ended reads. Raw sequencing data were processed using the HISAT2 (v2.2.1) alignment workflow [24]. Clean reads were mapped to the strawberry reference genome to quantify the transcript abundance. Differential expression analysis was performed with “DESeq2” package in the R language (v4.3.1), with significantly differentially expressed genes (DEGs) identified based on an adjusted q-value (FDR) < 0.05 and an absolute log2 fold change > 1 [25]. A Gene Ontology (GO) enrichment analysis of the DEGs was conducted using the “clusterProfiler” package within TBtools (v2.0), and the top 20 most significantly enriched GO terms were visualized [26].

### 2.6. Root Metabolite Composition Analysis

Strawberry seedlings were grown in a sterilized, soil-free substrate composed of vermiculite and perlite (1:1, *v*/*v*) and irrigated with sterile Hoagland nutrient solution. After 15 days of growth under each treatment, as indicated, the rhizosphere culture solution was collected, filtered through 0.22 μm membranes to remove microbial cells, and then freeze-dried. Lyophilized samples were analyzed via ultra-high-performance LC-MS (Vanquish UHPLC, Thermo, Waltham, MA, USA) to identify and quantify metabolites. Metabolites were tentatively annotated by matching the measured *m*/*z* values and MS/MS spectra to the KEGG Compound database. Partial least-squares-discriminant analysis (PLS-DA) was performed in R to discriminate metabolite profiles across treatments. To identify the key metabolites that are associated with root growth promotion, a random forest (RF) model was implemented using the “rfPermute” R package (v2.5.5) [27]. Fresh root weight was designated as the response variable, while metabolite abundances served as predictors. Key metabolites were ranked based on their contribution to model performance, specifically the increase in the mean squared error (IncMSE), when each variable was permuted. Partial dependence plots (PDPs) were generated using the random forest model to assess the relationship between individual metabolite abundances and root fresh weight. Metabolite abundance data were obtained from an LC-MS-based rhizosphere profiling, and the analysis accounted for the average effect of all other variables in the model [28]. To evaluate the robustness and relative contribution of the top-ranked metabolites, a secondary RF model was built using only the five most important features. In this model, predictors were iteratively excluded—first one at a time and then in pairs—and the model’s accuracy was assessed to identify features with the greatest impact on predictive performance [29].

To further validate the biological effect of the key metabolites identified through a random forest analysis, an exogenous application experiment was conducted. Strawberry seedlings were first grown under controlled conditions for 15 days, after which they were subjected to the same *Trichoderma* inoculation treatments as described above. In parallel, the selected metabolite was applied exogenously at a final concentration of 100 μM based on recommendations from a previous study [30]. The root development parameters and *Trichoderma* colonization levels were assessed as described above.

### 2.7. Statistical Analysis

The data collected consisted of the means from three replicates, and statistical analysis was conducted using SPSS 13.0 (SPSS Inc., Chicago, IL, USA). One-way ANOVA was used to compare multiple treatment groups when a single factor was involved. For experiments involving two independent variables, a two-way ANOVA was conducted to assess both individual and interaction effects. Statistical significance was set at *p* < 0.05.

## 3. Results

### 3.1. TgSWO-Overexpressing Trichoderma Improves the Growth of Strawberry Seedlings and Field Seedlings

**Figure 1:**
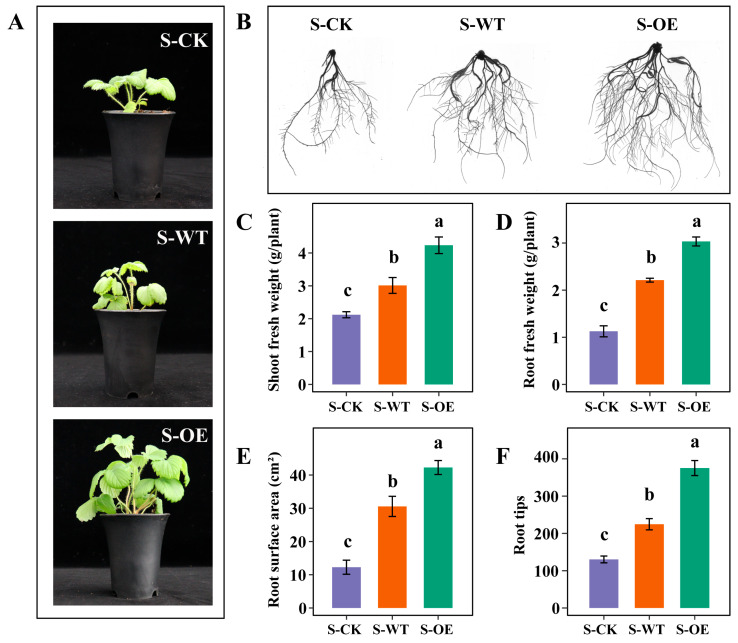
Effects of *Trichoderma* strain inoculation on the growth of strawberry seedlings. (**A**) Phenotypic comparison of strawberry seedlings after 30 days of treatment under the following three conditions: uninoculated control (S-CK), inoculation with the WT strain (S-WT), and inoculation with the OE strain (S-OE). (**B**) The root morphology of the corresponding seedlings is shown in panel A, illustrating the impact of each treatment on the root system’s development. (**C**–**F**) Quantitative analysis of the shoot fresh weight (**C**), root fresh weight (**D**), root surface area (**E**), and number of root tips (**F**) in the strawberry seedlings from panel (**B**). Error bars represent the standard error (SE, *n* = 3). Different letters indicate statistically significant differences among treatments based on one-way ANOVA (*p* < 0.05). WT: wild-type *Trichoderma guizhouense* NJAU4742; OE: *TgSWO*-overexpressing *Trichoderma guizhouense* NJAU4742; S-CK: uninoculated strawberry seedlings; S-WT: strawberry seedlings inoculated with the WT strain; S-OE: strawberry seedlings inoculated with the OE strain.

To investigate the effects of *TgSWO* gene overexpression in *Trichoderma* on strawberry growth, strawberry seedlings were treated with the wild-type *Trichoderma guizhouense* NJAU4742 and *TgSWO-overexpressing Trichoderma guizhouense NJAU4742* (OE) strains. As shown in Figure 1A,B, the strawberry inoculated with the OE strain (S-OE) exhibited more vigorous shoot and root growth compared with the strawberry inoculated with the WT strain (S-WT) or uninoculated strawberry seedlings (S-CK). This observation is corroborated by the quantitative analyses presented in Figure 1C–F. Specifically, S-OE displayed a significantly greater shoot fresh weight and root fresh weight than S-WT (1.41- and 1.37-fold increases, respectively) and S-CK (2.00- and 2.69-fold increases, respectively) (Figure 1C,D). Moreover, S-OE exhibited a notable increase in root surface area (1.38- and 3.44-fold compared with S-WT and S-CK, respectively) and the number of root tips (1.37- and 2.88-fold compared with S-WT and S-CK, respectively), indicating enhanced root development (Figure 1E,F). Taken together, these findings suggest that overexpression of the *TgSWO* gene markedly promotes plant growth and improves root system architecture.

**Figure 2:**
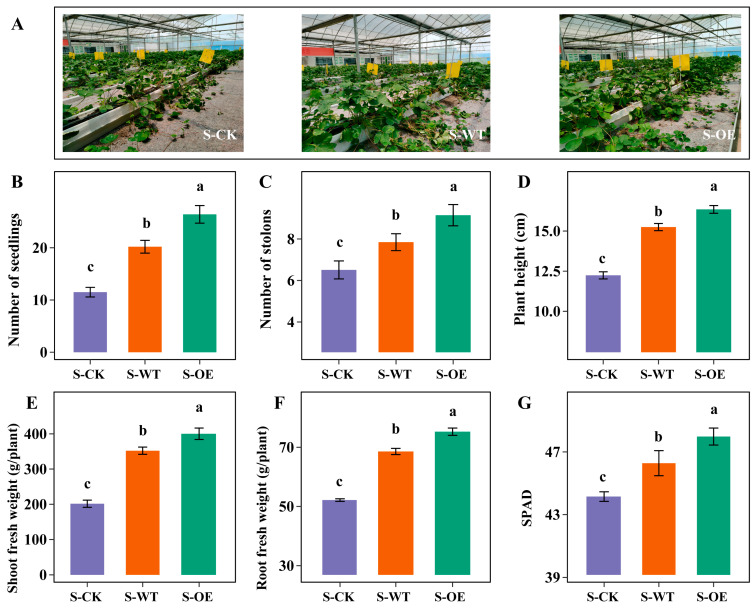
Effects of wild-type and *TgSWO*-overexpressing *Trichoderma* strains on the growth of strawberry under field conditions: (**A**) phenotype analysis of strawberry grown under field conditions and treated with wild-type and *TgSWO*-overexpressing *Trichoderma* strains; (**B**) number of strawberry seedlings; (**C**) number of stolons; (**D**) plant height; (**E**) shoot fresh weight; (**F**) root fresh weight; (**G**) SPAD of strawberry seedlings grown under field conditions after treatment with wild-type and *TgSWO*-overexpressing *Trichoderma* strains. The error bars in (**B**–**G**) indicate the SE (*n* = 3). Different letters in (**B**–**G**) indicate a significant difference compared with the control, as determined by one-way analysis of variance (ANOVA) at *p* < 0.05.

To further evaluate the impact of *TgSWO* overexpression on field-grown strawberry plants, additional agronomic traits were investigated under field conditions. As shown in Figure 2, compared with S-CK and S-WT, S-OE exhibited a markedly enhanced growth performance. S-OE produced a significantly greater number of strawberry seedlings and stolons than those in the S-CK and S-WT groups (Figure 2B,C). Moreover, S-OE displayed increased plant height, elevated SPAD (soil plant analysis development) values indicative of higher chlorophyll content, and significantly greater shoot and root fresh weights (Figure 2D–G). Collectively, these results demonstrate that *TgSWO* overexpression also promotes vegetative propagation and overall biomass accumulation in strawberry.

### 3.2. TgSWO-Overexpressing Trichoderma Significantly Affects the Root Colonization and Root Architecture

**Figure 3:**
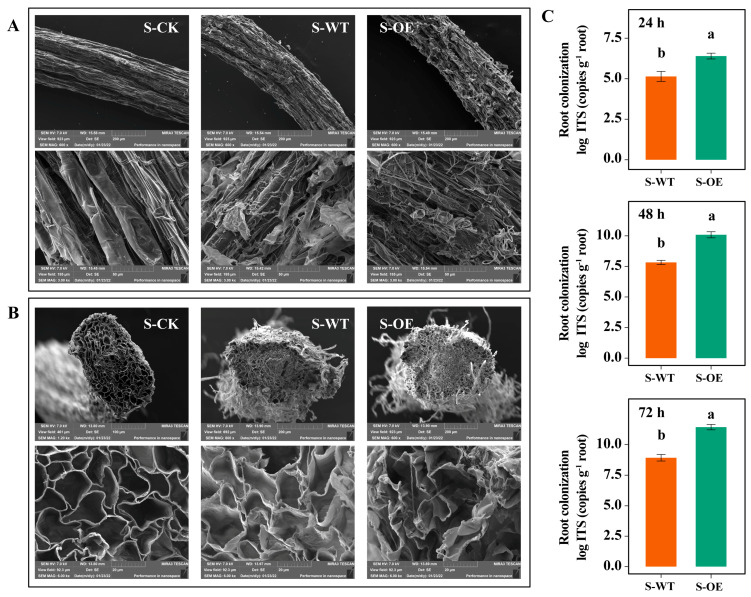
Effect of *Trichoderma* inoculation on root architecture and root colonization in strawberry seedlings: (**A**) SEM observation of strawberry seedling roots under the following three conditions: S-CK, S-WT, and S-OE; (**B**) SEM observation of the root cell morphology from the corresponding samples in panel (**A**); (**C**) quantitative analysis of *Trichoderma* colonization in strawberry roots inoculated with the WT and OE strains using real-time fluorescence quantitative PCR. Error bars represent the standard error (SE, *n* = 3). Different letters indicate statistically significant differences among treatments based on one-way ANOVA (*p* < 0.05). WT: wild-type *T. guizhouense* NJAU4742; OE: *TgSWO*-overexpressing *T. guizhouense* NJAU4742; S-CK: uninoculated strawberry seedlings; S-WT: strawberry seedlings inoculated with the WT strain; S-OE: strawberry seedlings inoculated with the OE strain.

To investigate root colonization by *Trichoderma* in strawberry, field-emission scanning electron microscopy (SEM) was employed to visualize hyphal accumulation in root tissues. As shown in Figure 3A, compared with the control group, hyphae were observed on the epidermis and outer cortex of strawberry roots in both the S-WT and S-OE groups. In the S-WT group, only a small number of hyphae were detected on the epidermis, with no hyphae present in the endodermis. In contrast, the S-OE group exhibited abundant hyphal colonization in both the epidermis and outer cortex. Notably, hyphal spores were also detected in the endodermis of roots treated with *TgSWO*-overexpressing *Trichoderma*. To further quantify colonization, quantitative PCR was performed to determine *Trichoderma* abundance in the roots. Consistent with the SEM observations, *Trichoderma* colonization in the S-OE group was significantly higher than that in the S-WT group. These results suggest that *TgSWO* overexpression substantially enhances *Trichoderma* colonization in strawberry roots.

Additionally, SEM was used to examine changes in root cell morphology following the OE treatment. As shown in Figure 3C, root endodermal cells in the S-OE group were noticeably larger and more loosely arranged compared to those in the WT group, indicating that *TgSWO*-overexpressing *Trichoderma* also alters root anatomical structure and architecture.

### 3.3. Transcriptomic Analysis Reveals TgSWO-Induced Molecular Mechanisms Underlying Trichoderma-Mediated Strawberry Growth Promotion

**Figure 4:**
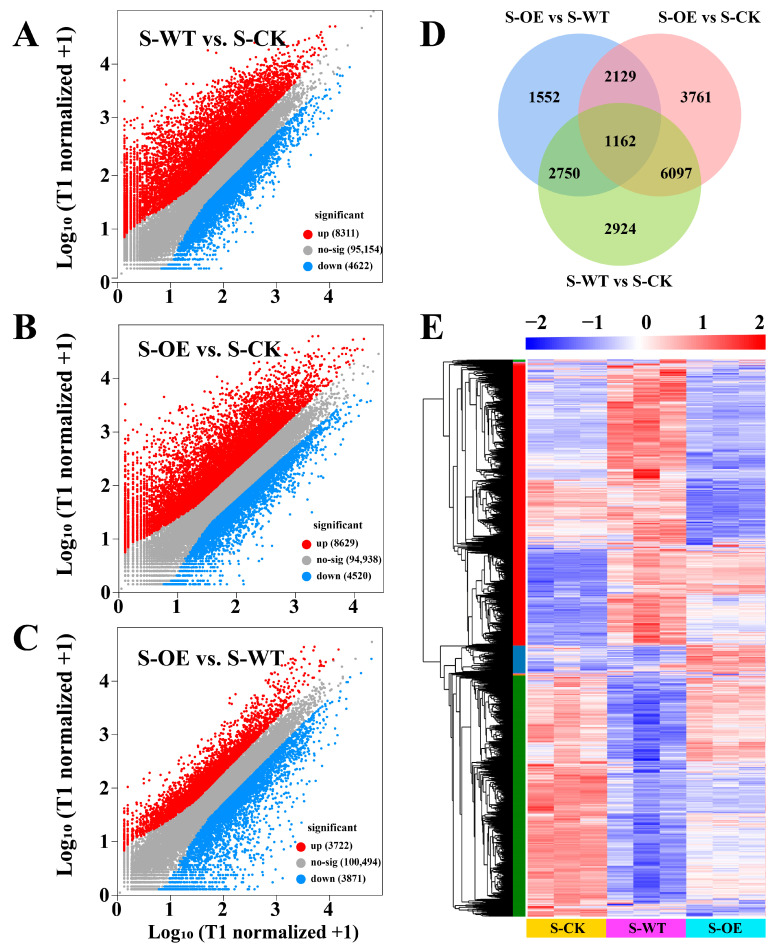
Overview of differentially expressed genes (DEGs) in strawberry roots across different treatments. (**A**–**C**) Volcano plots showing DEGs among the following three comparisons: S-WT vs. S-CK, S-OE vs. S-CK, and S-OE vs. S-WT. The red dots indicate up-regulated genes, blue dots indicate down-regulated genes, and grey dots represent non-significant genes (|log_2_ fold change| > 1, FDR < 0.05). (**D**) Venn diagram illustrating the overlap of DEGs between different comparison groups. (**E**) Hierarchical clustering heatmap of DEGs across the S-CK (yellow), S-WT (purple), and S-OE (cyan) groups.

To investigate how *TgSWO*-overexpressing *Trichoderma* promotes strawberry root development, we performed a transcriptome analysis on 15-day-old strawberry roots in the S-CK, S-WT, and S-OE groups (Figure 4). Compared to S-CK, the S-WT treatment resulted in 12,933 differentially expressed genes (DEGs), with 8311 genes up-regulated and 4622 genes down-regulated (Figure 4A,D,E). Similarly, OE treatment led to 13,149 DEGs compared to CK, including 8629 up-regulated and 4520 down-regulated genes (Figure 4B,D,E). A direct comparison between OE and WT revealed 7593 DEGs, of which 3722 genes were up-regulated and 3871 genes were down-regulated (Figure 4C–E). The large number of DEGs between OE- or WT-treated plants indicates that *TgSWO* overexpression induces additional transcriptional reprogramming beyond the baseline response triggered by the wild-type *Trichoderma*. These results suggest that *TgSWO* not only amplifies the general growth-promotion mechanisms associated with *T. guizhouense* inoculation but may also activate distinct molecular pathways specifically contributing to enhanced root development.

**Figure 5:**
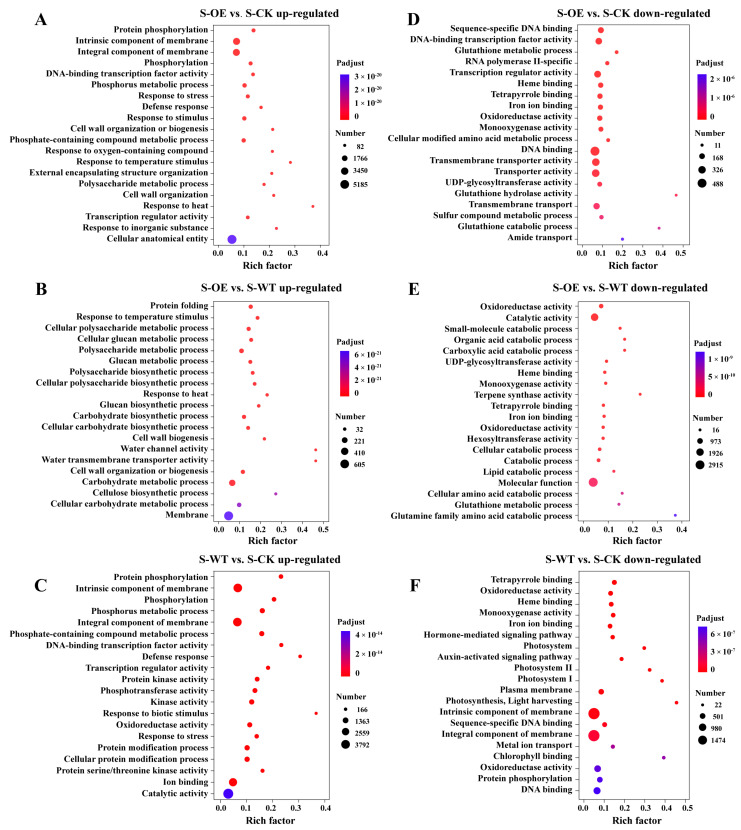
Gene Ontology (GO) enrichment analysis of the DEGs in the strawberry roots under different *Trichoderma* strain treatments: (**A**–**C**) GO enrichment of the up-regulated genes in S-OE vs. S-CK (**A**), S-OE vs. S-WT (**B**), and S-WT vs. S-CK (**C**); (**D**–**F**) GO enrichment of down-regulated genes in S-OE vs. S-WT (**D**), S-OE vs. S-CK (**E**), and S-WT vs. S-CK (**F**). The top 20 most significantly enriched GO terms (ranked by Padjust) are shown. The circle size indicates the number of associated genes, while the color represents the level of statistical significance.

To further explore these transcriptional changes, a GO enrichment analysis was performed on the DEGs (Figure 5). In both the S-WT and S-OE groups, compared with S-CK, a large number of overlapping GO terms were observed. These included protein phosphorylation (GO:0006468), phosphorus metabolic process (GO:0006793), DNA-binding transcription factor activity (GO:0003700), transcription regulator activity (GO:0140110), and response to stress (GO:0006950). These shared responses indicate that both wild-type and OE Trichoderma strains activate fundamental signaling, metabolic regulation, and stress adaptation pathways in the plant. Notably, the S-OE treatment induced additional specific enrichment in growth-related terms, such as cell wall organization or biogenesis (GO:0071554), polysaccharide metabolic process (GO:0005976), and external encapsulating structure organization (GO:0045229), which were not enriched in the WT group. These suggest a shift toward structural development in the S-OE roots. The S-OE vs. S-WT comparison further highlighted the processes enhanced by TgSWO overexpression, including cell wall biogenesis (GO:0042546), cellulose biosynthetic process (GO:0030244), glucan metabolic process (GO:0006073), and water channel activity (GO:0015250). These unique enrichments indicate that OE specifically promotes pathways associated with cell wall strengthening, carbohydrate biosynthesis, and water transport.

Conversely, under both the S-WT and S-OE treatments compared with S-CK, several common GO terms were significantly down-regulated. These included oxidoreductase activity (GO:0016491), heme binding (GO:0020037), monooxygenase activity (GO:0004497), and iron ion binding (GO:0005506). These functions are often associated with redox reactions, defense response, and metabolic detoxification, suggesting that both strains reduce plant oxidative burden during colonization. In addition to the shared categories, the S-OE vs. S-CK comparison revealed the specific suppression of glutathione metabolism (GO:0006749), DNA binding (GO:0003677), and transmembrane transport (GO:0055085), which were less prominent in the WT. These terms indicate a broader repression of stress-related transcription and transport activity in the OE-treated roots. The S-OE vs. S-WT comparison further highlighted transcriptional down-regulation of genes involved in catabolic metabolism, including small-molecule catabolic process (GO:0044282), lipid catabolic process (GO:0016042), organic acid catabolism (GO:0016054), and amino acid catabolism (GO:0009063). Molecular functions, such as terpene synthase activity (GO:0010333), monooxygenase activity (GO:0004497), and UDP-glycosyltransferase activity (GO:0008194), were also suppressed. These pathways are commonly related to secondary metabolism and stress compound biosynthesis. These results indicate that while both WT and OE *Trichoderma* reduce the expression of genes associated with oxidative stress and catabolism, the OE strain imposes a broader metabolic reprogramming by strongly down-regulating energy-expensive defense and degradation pathways, possibly reallocating resources toward growth and structural development.

### 3.4. Root Metabolic Profiling Uncovers Cysteine Associated with TgSWO-Enhanced Strawberry Performance

**Figure 6:**
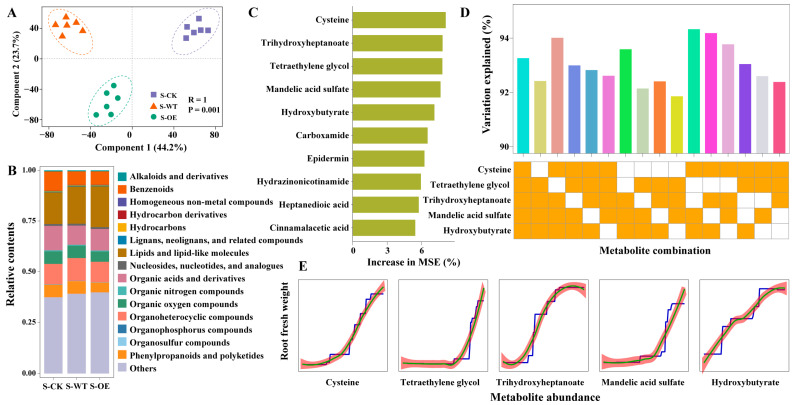
Metabolomics analysis of strawberry rhizosphere metabolites across different treatments: (**A**) PLS-DA score plot showing separation of metabolite profiles among treatments; (**B**) bar plot illustrating the classification of identified metabolites by category; (**C**) top metabolites associated with root promotion identified using a random forest model and ranked by the mean decrease in accuracy; (**D**) sparse random forest model of the top five predictive metabolites, and the grid below indicates whether each variable on the left was retained in the model (orange = retained; white = not retained); (**E**) partial dependence plots showing the relationship between the root fresh weight and metabolite abundance. The blue line represents the partial dependence estimate, the green line shows the locally weighted polynomial regression trend, and the red shaded area indicates the 95% confidence interval.

To investigate whether *TgSWO*-overexpressing *Trichoderma* affects the rhizosphere metabolites of strawberry root, untargeted metabolomics was performed. The PLS-DA showed clear separation among the S-CK, S-WT, and S-OE groups (R^2^ = 1, *p* = 0.001), indicating that fungal inoculation significantly altered rhizosphere metabolites (Figure 6A). The stack bar plot analysis revealed changes in metabolite categories (Figure 6B). Benzenoids were most abundant in S-CK, followed by S-WT and S-OE. In contrast, lipid-like compounds were more enriched in S-OE. Organic oxygen compounds, phenylpropanoids, and polyketides showed a decreasing trend from S-CK to S-OE. To identify the metabolites that were strongly associated with root growth, a random forest model was applied (Figure 6C). The top-ranking metabolites with the highest increase in the mean squared error (IncMSE), including cysteine, hydroxybutyrate, trihydroxyheptanoate, and mandelic acid sulfate, representing amino acids, lipids, and phenolic derivatives. To pinpoint the most critical compound, the top five metabolites were used to build predictive models, with one or two metabolites dropped in each iteration. Dropping cysteine alone led to the largest decrease in the model’s accuracy. When two metabolites were dropped, combinations involving cysteine and hydroxybutyrate or trihydroxyheptanoate had the strongest negative impact (Figure 6D). Partial dependence analysis revealed a strong, near-linear positive association between cysteine abundance and root fresh weight (Figure 6E), suggesting that increased cysteine levels are positively correlated with enhanced root development under the S-OE treatment.

### 3.5. Cysteine Application Further Promotes the Trichoderma Colonization and Strawberry Growth

**Figure 7:**
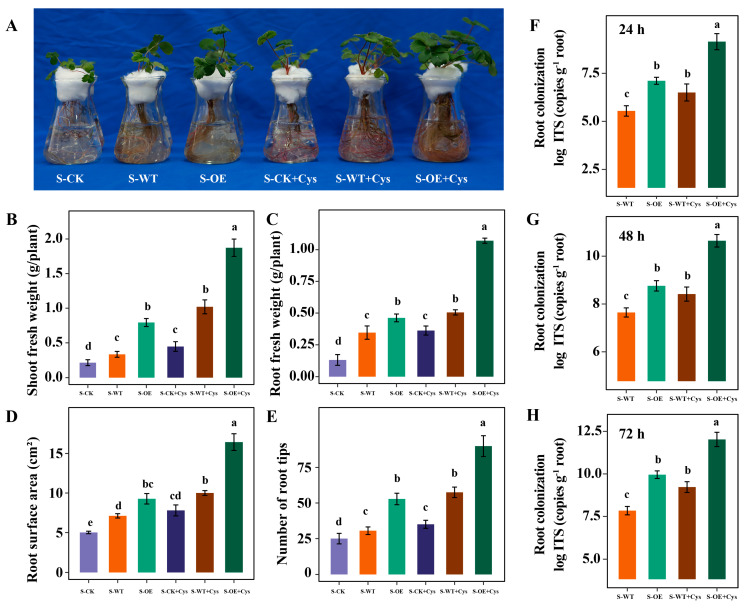
The effect of exogenous cysteine on the *Trichoderma* colonization and strawberry growth. (**A**) Phenotype analysis of strawberry seedlings treated with *Trichoderma* stain (wild-type or *TgSWO*-overexpressing *Trichoderma* strain) and exogenous cysteine. Analysis of shoot fresh weight (**B**), root fresh weight (**C**), root surface area (**D**), and root tips (**E**) of strawberry seedlings after treatment with a *Trichoderma* stain (wild-type or *TgSWO*-overexpressing *Trichoderma* strain) and exogenous cysteine. (**F**–**H**) Quantitative analysis of *Trichoderma* in strawberry seedlings treated with a *Trichoderma* strain (wild-type or *TgSWO*-overexpressing *Trichoderma* strain) and exogenous cysteine using real-time fluorescence quantitative PCR. The error bars in (**B**–**F**) indicate the SE (*n* = 3). Different letters in (**B**–**F**) indicate a significant difference compared with the control as determined by two-way analysis of variance (ANOVA) at *p* < 0.05.

Cysteine, a sulfur-containing amino acid, has been shown to enhance the growth of filamentous fungi and stimulate the secretion of lignocellulose-degrading enzymes, thereby promoting the degradation of plant cell walls and facilitating fungal colonization and nutrient cycling in the rhizosphere [17]. To investigate the role of cysteine in mediating the effects of the *TgSWO*-overexpressing strain on strawberry growth, cysteine supplementation experiments were conducted. S-CK, S-WT and S-OE were further treated with or without the addition of exogenous cysteine. Growth parameters, including shoot and root fresh weight, root surface area, and number of root tips, were recorded after a set cultivation period. As shown in Figure 7, both in the S-WT and S-OE groups, the application of exogenous cysteine significantly promoted strawberry growth, including both shoot and root development, as well as bacterial colonization. Notably, the growth-promoting effect of cysteine was most pronounced in the S-OE group. These findings suggest that cysteine application further promotes the *Trichoderma* colonization and strawberry growth.

## 4. Discussion

In this study, *Trichoderma* overexpressing *TgSWO* significantly promoted the growth of both strawberry seedlings and field-grown plants, demonstrating substantial agricultural implications. Firstly, from the perspective of plant growth, this provides a novel biological strategy for strawberry cultivation. Compared with traditional chemical fertilizers and pesticides, utilizing microorganisms and their functional genes is not only more environmentally friendly but also reduces potential chemical damage to the soil and ecosystem. Although previous studies have shown that certain *Trichoderma* species can promote plant growth [6], this is the first in-depth investigation into the role of the *TgSWO* gene in strawberry development.

The pronounced effects of *Trichoderma* overexpressing *TgSWO* on root colonization and root architecture further elucidate its potential mechanism in promoting plant growth. The root system, as a vital organ for water and nutrient uptake, is closely linked to plant health and development [31,32]. Enhanced root structure can improve nutrient absorption efficiency, and successful colonization by *Trichoderma* may alter the rhizosphere microenvironment by establishing a beneficial symbiotic relationship, thereby supporting overall strawberry growth [33]. These findings align with previous research demonstrating that interactions between beneficial microbes and plant roots influence plant growth, reinforcing the critical role of plant–microbe interactions in growth regulation.

The transcriptome results show that *TgSWO* overexpression leads to stronger activation of genes involved in cell wall biogenesis, polysaccharide metabolism, and membrane transport compared with both S-CK and S-WT. These genes are closely related to root cell expansion and lateral root formation [34], which is consistent with the observed increases in root length, surface area, and fresh weight in the S-OE group. Compared with the WT, OE specifically up-regulated genes related to cellulose and glucan biosynthesis, as well as water channel activity, suggesting *TgSWO* enhances the root-promoting effect beyond normal Trichoderma colonization. This may be due to its expansin-like function and its ability to stimulate signaling pathways like phosphorylation and transcriptional activation [6]. At the same time, OE down-regulated many genes involved in oxidative metabolism, glutathione-related redox processes, and small molecule catabolism. These pathways are often associated with plant stress responses and energy-consuming detoxification mechanisms [35]. The suppression of these processes suggests that *TgSWO* may help the plant reallocate metabolic resources away from defense and degradation toward growth-related functions. This reflects a typical growth-defense trade-off strategy, where reduced investment in protective pathways allows for enhanced biomass accumulation under favorable symbiotic conditions [36]. Such a shift likely contributes to the superior root development observed in the OE-treated plants.

While the benefits of *Trichoderma* in promoting plant growth are well established, recent studies have emphasized that the relationship between plants and fungi is bidirectional [37,38]. Not only does *Trichoderma* enhance plant growth, but plant root exudates also play a crucial role in promoting fungal colonization. Roots secrete various compounds, including auxins, sugars, and amino acids, which can directly influence the growth and behavior of beneficial microbes like *Trichoderma* [39]. In particular, auxins released by plant roots can induce the growth of the fungus, enhancing its ability to colonize the root system [40]. This reciprocal interaction creates a positive feedback loop, where Trichoderma facilitates plant growth, and, in turn, the plant’s own biochemical signals help establish a stable fungal community in the root zone. However, the molecular mechanisms behind it remain largely unexplored. The close association between cysteine and *TgSWO*-enhanced strawberry growth, as revealed by the root metabolomic profiling, represents a key highlight of this study. Previous research has demonstrated that cysteine directly promotes *Trichoderma* growth. Exogenous supplementation of cysteine significantly increases both the abundance and activity of *Trichoderma*, thereby creating more favorable conditions for subsequent root colonization [17,41]. Experimental results further confirmed that cysteine application not only enhanced the colonization capacity of *Trichoderma* in the strawberry rhizosphere but also promoted robust strawberry plant growth (Figure 7). This finding underscores the critical role of cysteine in this biological process. Among the altered metabolites, cysteine emerged as the most critical compound associated with increased root fresh weight (Figure 6). It is, therefore, reasonable to propose that *TgSWO* overexpression in *Trichoderma* stimulates the host plant to secrete more cysteine into the rhizosphere. This, in turn, may boost fungal growth and metabolic activity, forming a positive feedback loop, as follows: plant-derived cysteine promotes fungal colonization, and enhanced colonization further improves plant growth—both through physical modification of the root cell wall and the release of fungal effectors such as *TgSWO*. In addition, elevated cysteine levels may contribute to redox homeostasis and nutrient mobilization in the rhizosphere. As a precursor of glutathione and other thiol-containing compounds, cysteine plays a key role in mitigating oxidative stress, a factor known to influence root development and plant resilience [42].

Of note, a potential ecological consideration is that overexpression of *TgSWO* in *Trichoderma* may enhance the strain’s ability to modify plant cell walls and the rhizosphere environment. While this trait could promote strawberry root growth by facilitating root cell expansion, it might also create conditions that inadvertently benefit other fungal pathogens capable of exploiting similar cell-wall-degrading enzymes. For instance, plant pathogenic fungi such as *Fusarium* and *Botrytis cinerea* can utilize expansins or related cell wall hydrolases to invade host tissues [43,44]. Elevated expansin levels in the rhizosphere could unintentionally promote their colonization or virulence, depending on the specific microbial community and environmental context. Conversely, *Trichoderma* strains overexpressing *TgSWO* may also bolster the plant’s overall resistance to pathogens, with the positive effects likely outweighing any potential indirect risks associated with expansin secretion. Nevertheless, this theoretical possibility underscores the need for future studies to assess the strain’s effects on rhizosphere microbial communities—particularly pathogenic fungi—under controlled greenhouse conditions or in field trials. Such evaluations are crucial to ensure the safe and sustainable use of this transgenic *Trichoderma* strain in agricultural systems.

## 5. Conclusions

In summary, this study reveals the potential of *Trichoderma* overexpressing *TgSWO* in promoting strawberry growth and offers valuable insights for advancing microbial biotechnology in agriculture. From a practical perspective, this discovery opens a new avenue for improving strawberry cultivation. Precise regulation of cysteine application is expected to be an effective strategy to enhance *Trichoderma* colonization. By leveraging cysteine’s ability to stimulate *Trichoderma* growth, the synergistic effects of both agents can be harnessed to achieve more efficient and high-quality strawberry production. While associations between *Trichoderma* overexpressing *TgSWO* and strawberry growth were established, the underlying mechanisms require further elucidation.

## Data Availability

The original contributions presented in this study are included in the article. Further inquiries can be directed to the corresponding authors.
